# Maternal Healthcare Access and Childbirth Outcomes in Rural, Resource-Limited Settings: Evidence from the Eastern Cape, South Africa

**DOI:** 10.3390/ijerph23060700

**Published:** 2026-05-25

**Authors:** Aphilile Mdume, Kedibone Petunia Maake, Zisiwe Mahlati, Xolelwa Ntlongweni

**Affiliations:** 1 School of Public Health, Walter Sisulu University, Mthatha 5117, South Africa; aphililemdume@gmail.com (A.M.); kmaake@wsu.ac.za (K.P.M.); zmahlati@wsu.ac.za (Z.M.); 2 WSU Institute for Clinical Governance & Healthcare Administration, East London 5247, South Africa; 3 Global Centre for Human Resources for Health Intelligence, Walter Sisulu University, East London 5247, South Africa; 4 WSU Society and Health Research Institute, Walter Sisulu University, Mthatha 5117, South Africa

**Keywords:** antenatal care, childbirth outcomes, maternal healthcare access, neonatal health, rural health, women’s health, South Africa

## Abstract

**Highlights:**

**Public health relevance—How does this work relate to a public health issue?**

- Maternal healthcare utilization was high in this rural South African setting, yet adverse childbirth outcomes persisted.
- It examines whether high reported service utilization translates into improved childbirth outcomes in a resource-limited setting.

**Public health significance—Why is this work of significance to public health?**

- Conventional indicators of maternal healthcare access, including antenatal care attendance and facility-based delivery, were not statistically associated with childbirth outcomes.
- Delayed initiation of antenatal care emerged as a critical gap that may limit the effectiveness of subsequent care.

**Public health implications—What are the key implications or messages for practitioners, policy makers and/or researchers in public health?**

- Maternal health strategies should prioritize early antenatal booking, quality of care, and system-level interventions beyond service coverage indicators to improve neonatal outcomes.
- Strengthening the timing and effectiveness of care is essential for improving neonatal outcomes in rural and resource-limited settings.

**Abstract:**

Background: Maternal healthcare access is a critical determinant of women’s and neonatal health, especially in rural areas of low- and middle-income countries. Despite high reported utilization in South Africa, rural communities continue to experience adverse childbirth outcomes. Objective: To evaluate the association between maternal healthcare access and childbirth outcomes in Ingquza Hill Local Municipality, Eastern Cape Province of South Africa. Methods: A cross-sectional study was conducted among 213 pregnant and postpartum women receiving maternal healthcare services at St Elizabeth Hospital, a regional referral hospital serving multiple primary healthcare clinics across the municipality. Data were collected using structured questionnaires and maternity access indicators (including ANC attendance, timing of first ANC visit, number of visits, physical accessibility, and place of delivery), and childbirth outcomes. Logic regression analyses were performed to identify associations between access indicators and unfavorable childbirth outcomes. Results: Utilization of maternal healthcare services was high, with 96% of participants reporting ANC attendance, 92% receiving skilled care during pregnancy, and 91% delivering in a health facility. Unfavorable childbirth outcomes were observed in 12% of participants. Conventional indicators of maternal healthcare access, including ANC attendance, number of visits, physical accessibility, and place of delivery, were not statistically associated with childbirth outcomes in regression analyses. However, initiation of antenatal care was notably delayed, with a mean gestational age at first ANC visit of 21.7 weeks. The limited number of adverse outcomes constrained statistical power to detect modest associations. Conclusions: High maternal healthcare utilization alone did not ensure improved childbirth outcomes in this rural setting. Delayed initiation of antenatal care emerged as a critical gap that may limit the effectiveness of subsequent care, highlighting the limitations of coverage-based access indicators. Strategies to improve maternal and neonatal outcomes should prioritize early antenatal engagement, quality and continuity of care, and move beyond utilization metrics toward measures of effective coverage in rural and resource-limited contexts.

## 1. Introduction

Maternal and child health remains a central public health priority globally and is widely recognized as a key indicator of health system performance and equity in healthcare delivery [1,2], despite notable global progress, pregnancy and childbirth in low- and middle-income countries (LMICs). An estimated 178,000 maternal deaths and 1 million newborn deaths occur annually in the African region, with progress toward the Sustainable Development Goals (SDGs) target of less than 70 maternal deaths per 100,000 live births by 2030 considered insufficient without accelerated interventions [1,3]. Although the region has achieved reductions in maternal and neonatal mortality over the past two decades, it still accounts for 70% of global maternal death and nearly half of all newborn deaths and stillborns [1,3].

Access to maternal healthcare is multidimensional, encompassing availability, geographic accessibility, affordability, acceptability, and quality of services [2,4]. Women’s ability to seek and use necessary healthcare during pregnancy and childbirth is shaped by these interrelated factors. Physical barriers such as long distances to facilities, lack of reliable transport, and associated financial constraints continue to impede access for many rural women in Sub-Saharan Africa (SSA), even where services are provided free of charge [2,4]. Moreover, delayed engagement with care, especially late initiation of antenatal care (ANC), is a persistent challenge in SSA, with regional analyses indicating that over 60% of women delay first ANC visits beyond the first trimester, a factor consistently associated with poorer maternal and neonatal outcomes [4,5].

South Africa has implemented a national policy of free public maternal healthcare, with broad coverage of core services including ANC, skilled birth attendance, and facility-based deliveries. National data show generally high utilization rates for these two services; however, disparities in maternal mortality persist across provinces and between urban and rural settings. For example, the latest District Health Barometer indicates that while the national in-facility maternal mortality ratio (iMMR) was approximately 100.6 per 100,000 live births in 2023/2024, only a few provinces met the SDG targets, and the Eastern Cape continues to record significantly higher mortality compared to other provinces [6], reflecting inequities in access and outcomes. Similarly, Eastern Cape women are reported to be twice as likely to die from pregnancy or childbirth-related causes compared with women in provinces such as the Western Cape, highlighting persistent regional disparities in maternal health outcomes [6,7].

Beyond mortality, South African health surveillance reveals concerning trends in service utilization and quality of care. Recent reports have documented declines in ANC attendance at a national level, with the percentage of women attending any antenatal visit falling from 76.4% to 69.4% between 2022/23 and 2023/24, and declines observed in 50 of 52 districts [6]. Structural challenges, including understaffed facilities, transport barriers for rural women, and shortages of essential equipment, have been noted particularly in Eastern Cape maternity wards, undermining care quality and potentially contributing to adverse outcomes [7,8].

The discrepancy between high maternal healthcare utilization rates and ongoing adverse health outcomes has also been observed in the broader SSA context. Empirical evidence suggests that while ANC attendance and health facility delivery are associated with reduced neonatal mortality, the impact is often moderated by the timing of care initiation and quality of services, with incomplete or uninterrupted care associated with a limited protective effect [5,9]. Additionally, system-level barriers and social determinants such as low educational attainment, socioeconomic disadvantage, and inadequate community support continue to shape maternal health behaviours and outcomes across SSA, reducing the effectiveness of access-focused interventions when implemented in isolation [2,9].

Despite the established importance of healthcare access, less is understood about how conventional access indicators (such as distance to facilities, transport mode, and number of ANC visits) correlate with childbirth outcomes in rural South African contexts, where utilization appears relatively high [3,10]. Existing research highlights this gap and emphasizes the need for context-specific evidence to inform strategies that address not only coverage but also timelines, quality, and continuity of maternal care [3,10]. By focusing on Ingquza Hill local municipality, a predominantly rural area within the Eastern Cape Province, this study examines the association between maternal healthcare access and childbirth outcomes, aiming to clarify whether current service delivery strategies yield measurable benefits for neonatal health and identify opportunities for targeted intervention.

The study aimed to examine the association between maternal healthcare access and childbirth outcomes among pregnant and postpartum women in Ingquza Hill Local Municipality, with a focus on identifying gaps in service utilization, timing, and quality that may affect neonatal health.

## 2. Materials and Methods

A cross-sectional study was conducted at St Elizabeth Hospital, the primary referral hospital serving 32 wards in Ingquza Hill Local Municipality, OR Tambo District, Eastern Cape. The facility receives maternity referrals from multiple primary healthcare clinics and rural communities across 32 wards. The facility provides antenatal, intrapartum, postnatal, and neonatal services to a dispersed rural population.

### 2.1. Study Population and Sampling

Participants were recruited during routine antenatal, intrapartum, and postnatal care visits at St Elizabeth Hospital. Stratified random sampling was used to ensure representation across wards within Ingquza Hill Local Municipality. The final sample comprised 213 participants, accounting for a 105 non-response adjustment.

#### 2.1.1. Inclusion Criteria

i. Participants were women of reproductive age, defined as 18–49 years, who were receiving maternity services at St Elizabeth Hospital during the study period.
ii. Eligible participants were recruited from various maternal healthcare service points within the facility, including the antenatal care unit, women admitted in active labor, and postpartum women who had delivered at the institution.
iii. Women who delivered at home or en route to St Elizabeth Hospital and were subsequently admitted for postnatal care.

The selected age range reflects the standard reproductive age group and aligns with national and international maternal health guidelines.

#### 2.1.2. Exclusion Criteria

i. If the woman’s health status did not involve pregnancy, childbirth, or postnatal care
ii. If they were admitted to non-maternity healthcare services.
iii. Women who were unable to provide informed consent or who declined participation.

### 2.2. Data Collection

Data were collected using structured interviewer-administered questionnaires in English or IsiXhosa. Questions covered: sociodemographic (age, marital status, employment, education); Maternal healthcare access (distance to facility, transport, ANC attendance, skilled care, place of delivery, perceived quality); Childbirth outcomes (favorable/unfavorable neonatal health indicators at birth). Participants were recruited by trained research staff during pregnancy and in the early postpartum period while attending antenatal, delivery, or postnatal services at St Elizabeth Hospital. To minimize recall bias, information on childbirth outcomes was primarily obtained from maternity and neonatal health records, including birth status and immediate neonatal indicators. Self-reported data were used mainly for sociodemographic characteristics and healthcare access experiences.

### 2.3. Measures

Maternal healthcare access was assessed across multiple dimensions, including physical accessibility (distance to healthcare facility, mode of transport), utilization (antenatal care attendance, number of ANC visits, skilled care during pregnancy, and place of delivery), and perceived quality of care.

Childbirth outcomes were classified as favorable or unfavorable based on neonatal health indicators recorded in maternity records, including birth status and immediate health complications.

Unfavorable childbirth outcomes included indicators of compromised neonatal health at birth, such as low birth weight, need for immediate neonatal intervention, birth complications documented in maternity records, or adverse neonatal status at delivery.

This study used a structured, researcher-developed questionnaire informed by established maternal healthcare access frameworks and national maternal health indicators. The instrument was designed to capture key dimensions of healthcare access and utilization throughout pregnancy, childbirth, and the postnatal period, as well as childbirth outcomes. Questionnaire development was guided by a review of the literature and alignment with indicators commonly used in maternal and neonatal health research.

The final questionnaire consisted of approximately 30 items; all of which were included in the analysis.

The questionnaire was piloted among a small group of women receiving antenatal and maternity services to assess clarity, acceptability, and completeness. Content validity was supported through a review of the literature and consultation with maternal healthcare professionals. The instrument demonstrated good internal consistency (Cronbach’s alpha = 0.84) across items included in the analysis.

### 2.4. Statistical Analysis

Data were analyzed using SPSS v29.0. Descriptive statistics summarized the participants and access measures. Univariable logistic regression identified associations between access indicators and childbirth outcomes. Variables with *p* < 0.2 in univariable analysis or clinical relevance were included in multivariable models. Statistical significance was set at *p* < 0.05. Childbirth outcomes were analyzed as a binary variable (favorable versus unfavorable), and associations with maternal healthcare access indicators were examined using logistic regression models.

### 2.5. Ethical Considerations

Ethical approval for the study was obtained from Walter Sisulu University Human Research Ethics Committee (HREC/099/2025) and the Eastern Cape Department of Health. Written informed consent was obtained from all participants prior to participation. Participants were informed about the study objectives, procedures, potential risks and benefits, and their right to decline or withdraw at any time without consequence. Confidentiality and anonymity were strictly maintained.

## 3. Results

A total of 213 women participated in the study. Childbirth outcomes were classified as either favorable or unfavorable based on the neonatal health indicators recorded at birth. Of the 213 participants, 26 (12%) experienced unfavorable childbirth outcomes, while 187 (88%) had favorable outcomes.

### 3.1. Sociodemographic Characteristics of Participants

No statistically significant differences were observed between women with favorable and unfavorable childbirth outcomes across sociodemographic characteristics, including age, marital status, education level, employment status, and household income (Table 1). Utilization of maternal healthcare services was high across all assessed dimensions. Nearly all participants reported attending antenatal care during pregnancy (96%), the majority received care from a skilled health professional (92%), and most delivered in a healthy facility (91%) (Table 1).

Despite high utilization, initiation of antenatal care was notably delayed. The mean gestational age at first antenatal visit was 21.7 weeks, exceeding national and World Health Organization recommendations for first-trimester booking. Just over half of participants (51%) attended four or more ANC visits, while 49% attended fewer than four visits.

The distribution of unfavorable childbirth outcomes was similar across categories of ANC attendance, number of visits, skilled care during pregnancy, and place of delivery. No statistically detectable differences were observed in crude or adjusted analyses (Table 2). However, the limited number of adverse outcomes constrained statistical power to detect modest associations.

### 3.2. Maternal Healthcare Access and Utilization

Utilization of maternal healthcare services was high across all measured dimensions. Almost all participants (96%) reported attending antenatal care during pregnancy, and 92% received care from a skilled healthcare professional. Facility-based delivery was reported by 91% of participants, indicating widespread use of institutional maternity services. Despite high service utilization, initiation of antenatal care was notably delayed. The mean gestational age at first antenatal visit was 21.7 weeks, exceeding national and World Health Organization recommendations for first-trimester initiation. Just over 51% reported attending more than four antenatal care visits, while 49% attended fewer than 4 visits (Table 1). This pattern was consistent across participants with both favorable and unfavorable childbirth outcomes. No statistically significant differences were detected in crude or adjusted analyses (Table 2). However, the small number of adverse outcomes limited the power to detect modest associations between the number of ANC visits and childbirth outcomes. The distribution of unfavorable childbirth outcomes was similar across categories of antenatal care attendance, number of ANC visits, skilled care during pregnancy, physical accessibility measures, and place of delivery, with no statistically significant differences (Table 1).

### 3.3. Physical Accessibility to Maternal Healthcare Services

Most participants resided within 5–10 km of the referral hospital (44%), followed by those living less than 5 km away (37%). Access to care was commonly facilitated through public transport (56%), while a substantial proportion of women accessed services on foot (41%). Distance to facility and mode of transport did not differ meaningfully between women with favorable and unfavorable childbirth outcomes. Meaning distance to the facility was not a statistically detectable association (Table 2).

### 3.4. Perceived Quality of Care

Perceived quality of maternal healthcare services was generally rated positively. Over half of the participants (51%) rated the quality of care as very satisfactory, while 29% rated it as satisfactory. Perceptions of quality of care did not differ significantly between mothers who gave birth to healthy and unhealthy babies.

### 3.5. Place of Delivery

Facility-based delivery was common, with 91% of mothers delivering at St Elizabeth Hospital. Smaller proportions were delivered at home (6%), at clinics (1%), or before arrival at a health facility (2%). Place of delivery was not significantly associated with childbirth outcomes.

### 3.6. Age Distribution

The median age of participants was 24 years, with ages ranging from 18 to 43 years. Age distribution was similar across childbirth outcome categories.

### 3.7. Factors Associated with Childbirth Outcomes

Univariable logistic regression analyses were conducted to examine associations between maternal sociodemographic characteristics, maternal healthcare access indicators, and unfavorable childbirth outcomes (Table 2). No maternal healthcare access indicators, including antenatal care attendance, number of visits, distance to facility, mode of transport, or place of delivery, were statistically significantly associated with childbirth outcomes.

Although selected variables, such as student status, showed elevated odds of unfavorable childbirth outcomes in crude analyses, these associations did not remain statistically significant after adjustment. Confidence intervals for several predictors were wide, reflecting limited precision due to the small number of adverse outcomes. In the multivariable logistic regression model, none of the included variables remained statistically significant predictors of unfavorable childbirth outcomes after adjustment for potential confounders (Table 3).

### 3.8. Multivariable Analysis and Key Findings

In the adjusted multivariable model, none of the included maternal healthcare access indicators emerged as independent predictors of childbirth outcomes. The absence of significant associations persisted after controlling for age, employment status, education level, and other relevant covariates.

### 3.9. Summary of Results

Taken together, the results indicate that while maternal healthcare utilization was high in this rural setting, conventional measures of access were not associated with improved childbirth outcomes. Delayed initiation of antenatal care emerged as a common pattern across the study population, highlighting a potential gap in the timing and effectiveness of care rather than its availability or utilization.

## 4. Discussion

This study examined the association between maternal healthcare access and childbirth outcomes among women in a rural municipality in the Eastern Cape of South Africa. Although utilization of antenatal care (ANC), skilled care during pregnancy, and facility-based delivery were high, conventional indicators of maternal healthcare access were not statistically associated with childbirth outcomes in regression analyses. These findings challenge the assumption that increased service utilization alone is sufficient to ensure improved neonatal health outcomes in rural and resource-limited settings, an assumption that continues to inform maternal health policy and monitoring frameworks in many low-and-middle-income countries [1,2]. The lack of statistically detectable associations observed in this study should not be interpreted as evidence that maternal healthcare access is unimportant. Instead, the findings highlight the limitations of commonly used access and utilization metrics, such as ANC attendance, number of visits, and place of delivery, when assessed without adequate consideration of timing, content, and effectiveness of care. Similar discrepancies between high service coverage and persistent maternal or neonatal morbidity and mortality have been documented across sub-Saharan Africa, where improvements in utilization have not consistently translated into expected health gains [5,9].

### 4.1. Timing for Antenatal Care as a Critical Determinant of Effective Coverage

One of the most important findings of this study was the delayed initiation of antenatal care, with the mean gestational age at first ANC visit occurring well beyond first-trimester recommendations. Early initiation of ANC is a cornerstone of effective maternal healthcare, as emphasized in World Health Organization (WHO) guidelines, which recommend first-trimester booking to facilitate timely risk identification, preventive interventions, and referral for complications [10]. Delayed entry into care compromises opportunities for early detection and management of maternal conditions such as hypertension, infections, anemia, and fetal growth restriction, all of which are associated with adverse neonatal outcomes.

In this context, the absence of statistically detectable differences in childbirth outcomes by number of ANC visits must be interpreted with caution. Visiting frequency alone, particularly when ANC begins late in pregnancy may be a weak proxy for effective coverage. Women who initiate care late may still accumulate several visits without receiving the full preventive and risk-reduction benefits associated with early engagement. This finding aligns with growing evidence suggesting that when care begins and what care is delivered are more critical determinants of maternal and neonatal outcomes than simple visit counts [1,9]. Studies examining the quality and content of ANC across sub-Saharan Africa have shown that even women who attend multiple visits may receive incomplete or suboptimal care, limiting the potential impact on birth outcomes [5,11,12].

### 4.2. Interpretation of Null Associations and Analytical Considerations

This study did not identify statistically significant associations between childbirth outcomes and sociodemographic characteristics, physical accessibility measures, or maternal healthcare utilization indicators. However, these findings must be interpreted in light of the relatively small number of adverse childbirth outcomes observed. The limited number of events constrained statistical power, increased estimate precision, and reduced the ability to detect modest but clinically meaningful associations, particularly in multivariable analyses. Wide confidence intervals across several predictors highlight the potential for Type II errors.

Accordingly, the regression findings are best viewed as exploratory rather than definitive. Importantly, the consistency of null findings across multiple conventional access indicators suggests that once minimum levels of physical access and service coverage are achieved, additional improvements in neonatal outcomes may depend more heavily on other dimensions of care, particularly their timeliness, community, and quality, than on utilization alone.

### 4.3. Rethinking Access-Focused Maternal Health Strategies

Traditional conceptualizations of maternal healthcare access emphasize physical availability and geographic accessibility, as articulated in influential frameworks such as the “three delays” model [4]. While these barriers remain critically important in many settings, the findings of this study suggest that most women resided within a reasonable distance of a healthcare facility, were able to access transport, and delivered within health institutions, which may partly explain the absence of statistically detectable associations between physical access measures and childbirth outcomes.

These findings reinforce the need to move beyond coverage-based indicators toward effective coverage frameworks, which integrate service utilization with the quality, content, and timeliness of care received [9,12]. Reliance on utilization metrics alone may overestimate health system performance and obscure persistent deficiencies in care delivery, particularly in rural health systems facing staff shortages, resource constraints, and variability in clinical practice [6,7]. Evidence from South Africa has repeatedly highlighted the role of health system quality, accountability, and responsiveness in shaping maternal and neonatal outcomes, even where services are nominally available [6,7,8].

### 4.4. Broader Public Health and Women’s Health Implications

From a public health perspective, these findings highlight the limitations of maternal health strategies that prioritize utilization targets without concurrent investment in early antenatal engagement and quality improvement. Interventions focused on promoting first-trimester ANC booking, strengthening the clinical content of initial visits, and improving continuity of care across the maternal health continuum may be particularly impactful in rural and resource-limited settings. Such interventions are consistent with national care guidelines in South Africa, which emphasize early booking and comprehensive risk assessment as foundations of quality maternal care [13].

The results also highlight the importance of addressing broader social and systemic determinants of maternal and neonatal health. Socioeconomic disadvantages, inequities in education and employment, and health system constraints may interact in complex ways to influence birth outcomes, even in the presence of high service utilization. Addressing these challenges will require integrated, multisectoral approaches that extend beyond the health system alone.

### 4.5. Implications for Future Research

Future research should prioritize larger, multisite studies that include recruitment from primary healthcare facilities and community settings, thereby capturing women who may not reach hospital-based services. Longitudinal designs would allow clearer assessment of causal pathways linking ANC timing, quality of care, and neonatal outcomes. Additionally, incorporating objective measures of care quality, such as service readiness assessments, clinical audits, and respectful maternity care indicators, alongside qualitative exploration of women’s experiences, would provide deeper insight into how maternal healthcare access translates into health impact in the rural South African context.

### 4.6. Study Limitations

This study has several limitations that should be considered when interpreting the findings. Participants were recruited exclusively from St Elizabeth Hospital, which may introduce selection bias and limit the generalizability of the results to women who access hospital-based maternity services. However, St Elizabeth Hospital functions as the main regional referral hospital for Ingquza Hill Local Municipality and receives patients referred from multiple primary healthcare clinics across the municipality’s 32 wards. As such, the study population reflects a broad rural catchment area rather than a single localized facility. Nevertheless, women who do not access facility-based services, including those delivering exclusively at home or in community settings, may be underrepresented.

The ANC visit frequency was examined, and a similar outcome distribution was observed, but we cannot rule out meaningful associations due to limited power, especially given delayed ANC initiation.

## 5. Conclusions

This study examined the relationship between maternal healthcare access and childbirth outcomes among women in a rural municipality in the Eastern Cape, South Africa. Despite high reported utilization of antenatal care, skilled care during pregnancy, and facility-based delivery, conventional measures of maternal healthcare access were not associated with improved childbirth outcomes. These findings indicate that broad service coverage, while necessary, is not sufficient to ensure favorable neonatal outcomes in rural and resource-limited settings.

A key insight from the study is the widespread delay in antenatal care initiation, with most women entering care well beyond the first trimester. Late engagement may limit the potential benefits of subsequent antenatal visits, reducing opportunities for early risk identification, preventive interventions, and timely referral. This pattern highlights an important gap between service availability and the delivery of timely and effective care.

The absence of statistically detectable associations between childbirth outcomes and commonly used access indicators further highlights the limitations of relying solely on utilization-based metrics to assess maternal healthcare performance. Once minimum levels of physical access and service uptake are achieved, improvements in neonatal outcomes may depend more strongly on the timing, quality, and continuity of care than on visit frequency or place of delivery alone.

From a public health and women’s health perspective, the findings support a shift toward maternal health strategies that emphasize early antenatal engagement, strengthened clinical content of care, and improved service quality across the continuum of care. Interventions that focus on effective coverage—integrating utilization with timeliness and quality—are likely to be particularly important in rural contexts characterized by structural and resource constraints.

Overall, this study highlights the need to move beyond coverage-focused indicators toward more comprehensive approaches to maternal healthcare that prioritize effectiveness, equity, and responsiveness. Addressing delays in care initiation and strengthening the quality of services are critical steps toward improving neonatal outcomes in rural and resource-limited settings.

## Figures and Tables

**Table 1:** Characteristics of mothers in the study.

Variables	Mothers Who Gave Birth to Unhealthy Babies (*n* = 26)	Mothers Who Gave Birth to Healthy Babies (*n* = 187)	Total (*n* = 213)	Chi Squared
Marital Status	n (%)	n (%)	n (%)	
Single	20 (77)	155 (83)	175 (82)	0.519
Married	6 (23)	29 (15)	35 (17)	
Widowed	0 (0)	3 (2)	3 (1)	
Education Level				
No formal	2 (8)	15 (8)	17 (5)	0.911
Primary	2 (8)	23 (13)	25 (12)	
Secondary	15 (57)	98 (52)	113 (53)	
Tertiary	7 (23)	51 (27)	58 (27)	
Employment				
Employed	1 (4)	10 (6)	11 (5)	0.126
Self-employed	2 (8)	2 (1)	4 (2)	
Student	4 (15)	38 (20)	42 (20)	
Unemployed	19 (73)	137 (73)	156 (73)	
Household income				
Less than R1000	3 (12)	12 (7)	15 (7)	0.272
R1000–R3000	6 (23)	79 (42)	85 (40)	
R3000–R5000	10 (38)	60 (32)	70 (33)	
More than R5000	7 (27)	36 (19)	40 (20)	
Distance to facility				
Less than 5 km	11 (42)	67 (36)	78 (37)	0.269
5–10 km	11 (42)	83 (44)	94 (44)	
More than 10 km	4 (16)	37 (20)	41 (19)	
Mode of transport				
Public transport	13 (50)	106 (57)	119 (56)	0.459
Walking	13 (50)	75 (40)	88 (41)	
Walking & public transport	0 (0)	6 (3)	6 (3)	
Clinic attendance				
No	1 (4)	7 (4)	8 (4)	0.979
Yes	25 (96)	180 (96)	205 (96)	
Quality of care				
Not applicable	2 (8)	8 (4)	10 (5)	0.670
Very dissatisfactory	4 (15)	16 (19)	20 (9)	
Dissatisfactory	1 (4)	13 (7)	14 (6)	
Satisfactory	6 (23)	55 (29)	61 (29)	
Very satisfactory	13 (50)	95 (51)	108 (51)	
Birthplace before arrival				0.761
Clinic	0	2 (1)	2 (1)	
Home	1 (4)	12 (7)	13 (6)	
St Elizabeth	25 (96)	169 (90)	194 (91)	
No. of ANC visits				
<4	12 (46)	92 (49)	104 (49)	0.771
>4	14 (54)	95 (51)	109 (51)	
Attended by a health worker				
No	1 (4)	15 (8)	16 (8)	0.449
Yes	25 (96)	172 (92)	197 (92)	

**Table 2:** Univariable logistic regression.

Variables	Unadjusted OR (95% CI)	*p*-Value
Age (years)	0.99 (0.93–1.05)	0.798
Marital status		
Single	Ref.	
Married	0.62 (0.23–1.07)	
Widowed	1	
Education Level		
No formal	1.14 (0.24–5.53)	0.863
Primary	1.76 (0.37–8.24)	0.473
Secondary	1.11 (0.42–2.91)	0.824
Tertiary	Ref.	
Employment status		
Employed	10 (0.58–171.20)	0.112
Self-employed	Ref.	
Student	9.5 (1.03–86.90)	0.046
Unemployed	7.21 (0.96–54.2)	0.055
Household income		
Less than R1000	0.78 (0.17–3.50)	0.743
R1000–R3000	2.56 (0.80–8.16)	0.112
R3000–R5000	1.17 (0.41–3.33)	0.774
More than R5000	Ref.	
Distance to facility		
Less than 5 km	Ref.	
5 km–10 km	1.24 (0.51–3.03)	0.639
More than 10 km	1.52 (0.45–5.11)	0.500
Mode of transport		
Public transport	Ref.	
Walking	0.71 (0.31–1.60)	0.410
Walking & Public transport	1	
Clinic attendance		
No	Ref.	
Yes	1.03 (0.12–8.71)	0. 979
Quality of care		
Not applicable	Ref.	
Very dissatisfactory	1 (0.15–6.67)	1
Dissatisfactory	3.25 (0.25–41.9)	0.366
Satisfactory	2.29 (0.39–13.37)	0.357
Very satisfactory	1.83 (0.35–9.55)	0.475
Birthplace before arrival		
Clinic	1	
Home	1.77 (0.22–14.25)	0.589
St Elizabeth	Ref.	
No. of visits		
<4	Ref.	
>4	0.88 (0.39–2.01)	0.771
Attended by HW		
No	Ref.	
Yes	0.46 (0.06–3.62)	0.460

OR—Odds Ratio; Ref—baseline category, the logistic regression used for comparison.

**Table 3:** Multivariable logistic regression.

Variables	Adjusted OR (95% CI)	*p*-Value
Age (years)	1.01 (0.94–1.10)	0.728
Education Level		
No formal	1.06 (0.17–6.49)	0.949
Primary	1.55 (0.30–7.99)	0.602
Secondary	1.04 (0.33.26)	0.943
Tertiary	Ref.	
Employment status		
Employed	5.42 (0.24–120.31)	0.285
Self-employed	Ref.	
Student	10.09 (0.71–141.39)	0.086
Unemployed	5.50 (0.53–57.41)	0.154
Household income		
Less than R1000	0.79 (0.14–4.32)	0.782
R1000–R3000	2.39 (0.64–8.98)	0.196
R3000–R5000	0.99 (0.31–3.14)	0.985
More than R5000	Ref.	
Distance to facility		
Less than 5 km	Ref.	
5 km–10 km	0.87 (0.26–2.83)	0.810
More than 10 km	1.01 (0.21–4.88)	0.990
Mode of transport		
Public transport	Ref.	
Walking	0.79 (0.25–2.49)	0.688
Walking & Public transport	1	
Clinic attendance		
No	Ref.	
Yes	0.94 (0.10–8.63)	0.958

OR—Odds Ratio. Ref—baseline category, the logistic regression used for comparison.

## Data Availability

The de-identified datasets generated and analyzed during this study are available from the corresponding author upon reasonable request. Data are securely stored on password-protected institutional servers and managed in accordance with ethical approvals and participant confidentiality requirements.

## Abbreviations

The following abbreviations are used in this manuscript:

UN	United Nations
MDG	Millennium Development Goals
SSA	Sub-Saharan Africa
GDP	Gross Domestic Product
SDG	Sustainable Development Goals
WHO	World Health Organization
HDI	Human Development Index
UNICEF	United Nations Children’s Fund
WDI	World Development Indicators
ANC	Antenatal care
PNC	Postnatal Care
ECDoH	Eastern Cape Department of Health
OR T	Oliver Reginald Tambo
NHI	National Health Insurance
SEH	St Elizabeth Hospital
StatsSA	Statistics South Africa
NVB	Normal Vaginal Birth
C/S	Caesarean Section
MPNH	Maternal, Perinatal, and Neonatal Health
LM	Local municipality
